# Open repair of a proximal left subclavian artery mycotic aneurysm with median claviculectomy

**DOI:** 10.1016/j.jvscit.2023.101374

**Published:** 2023-11-22

**Authors:** Ahmad Tabatabaeishoorijeh, Paul Haddad, Yusuf Chauhan, Marvin D. Atkins, Maham Rahimi

**Affiliations:** a School of Engineering Medicine, Texas A&M University, Houston, TX; b School of Medicine, Texas A&M University, Bryan, TX; c College of Engineering, Texas A&M University, Bryan, TX; d Department of Cardiovascular Surgery, Houston Methodist Hospital, Houston, TX

**Keywords:** Aneurysm, Claviculectomy, Great saphenous vein graft, Intravenous drug abuse, Mycotic, Subclavian artery aneurysm, Surgical technique

## Abstract

Surgical repair of a subclavian artery mycotic aneurysm is dependent on aneurysm-specific characteristics and anatomic exposures could require sternotomy, thoracotomy, or supraclavicular incisions. Alternatively, a median claviculectomy can be used. We successfully performed a subclavian artery to axillary artery bypass with median claviculectomy in a 23-year-old man with multiple comorbidities. Postoperative Doppler ultrasound showed a patent left axillary artery with a palpable left radial artery, and the patient demonstrated full left shoulder range of motion without any significant deformities. This case suggests that a median claviculectomy can produce satisfactory outcomes in patients with subclavian artery mycotic aneurysms.

Subclavian artery mycotic aneurysms (SAMAs) are extremely rare, accounting for <5% of the total subclavian artery aneurysms in the past 30 years.^1^^,^^2^ Although patients are often poor surgical candidates, more invasive methods of exposure such as thoracotomy or median sternotomy are still used in the treatment of SAMAs.^2^, ^3^, ^4^, ^5^, ^6^ An anatomic exposure using claviculectomy has remained underused and rather controversial. We report a median claviculectomy method of exposure for the successful repair of a 1.9-cm left SAMA distal to the left vertebral artery with a subclavian artery to axillary artery bypass using the right great saphenous vein. The patient provided written informed consent for the report of his case details and imaging studies.

## Case report

A 23-year-old man with a history of intravenous drug abuse had presented to an outside hospital with septic shock and multiorgan failure. At that time, he had mitral valve endocarditis secondary to *Streptococcus mitis* complicated by severe mitral regurgitation due to leaflet destruction. In addition to mitral valve endocarditis, diffuse septic embolic phenomena were observed, resulting in left subclavian artery occlusion with arm rest pain, which resolved with systemic anticoagulation. The endocarditis was treated surgically using a bioprosthetic mitral valve, and he completed a course of ceftriaxone monotherapy postoperatively before discharge.

**Fig 1:**
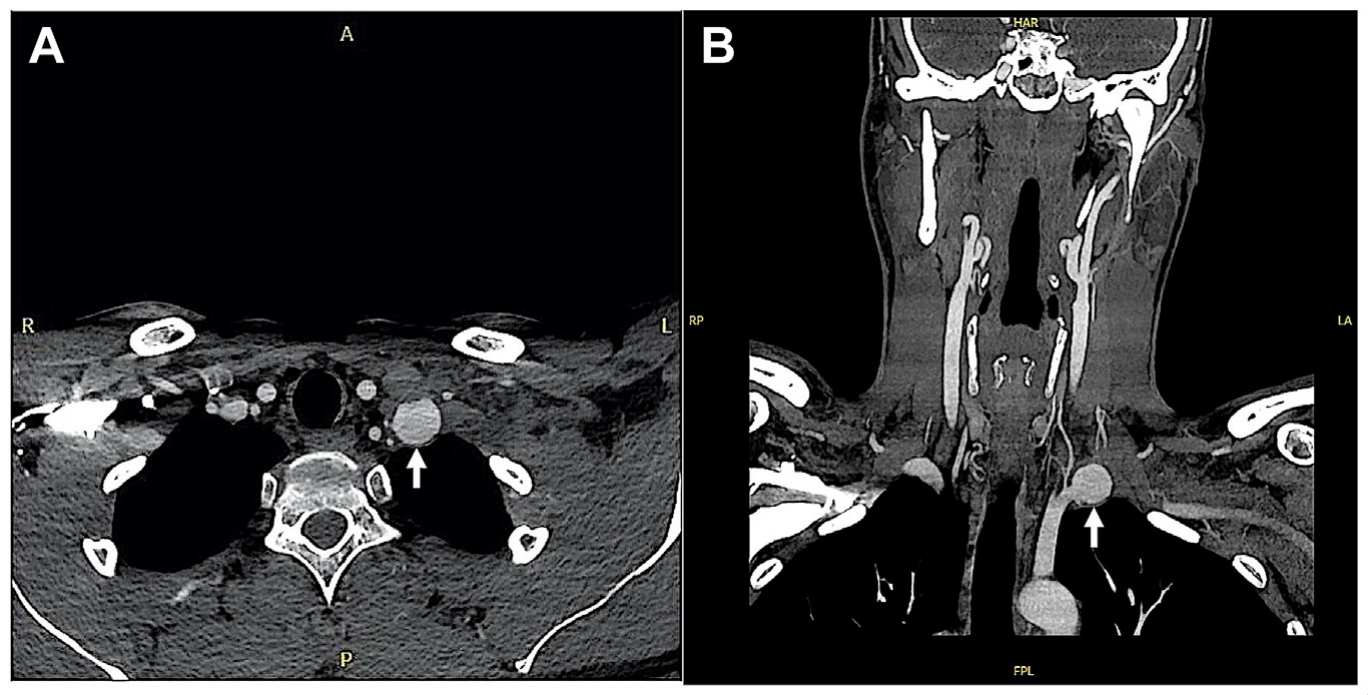
**a,** Axial view of computed tomography angiogram showing a 1.9 × 1.5-cm subclavian artery mycotic aneurysm (SAMA; *arrow*). **b,** Coronal view of computed tomography angiogram showing a 1.9 × 1.5-cm SAMA (*arrow*).

Four months later, the patient presented to an outside hospital emergency room with severe left hand weakness and pain. Computed tomography angiography of the left upper extremity revealed a 1.9 × 1.5-cm left subclavian artery mycotic aneurysm that caused a focal occlusion of the distal subclavian artery with reconstitution at the level of the axillary artery (Fig 1). The patient was transferred to our vascular surgery department where daptomycin was administered for antibiotic coverage and intravenous heparin was instituted for anticoagulation therapy before scheduled surgery. The patient's medical history and comorbidities were considered, and the risk, benefits, and alternatives of the various methods of exposure were discussed with the patient. The decision was made to perform left median claviculectomy due to the complex and unique anatomic exposure involving both intrathoracic and extrathoracic portions.

**Fig 2:**
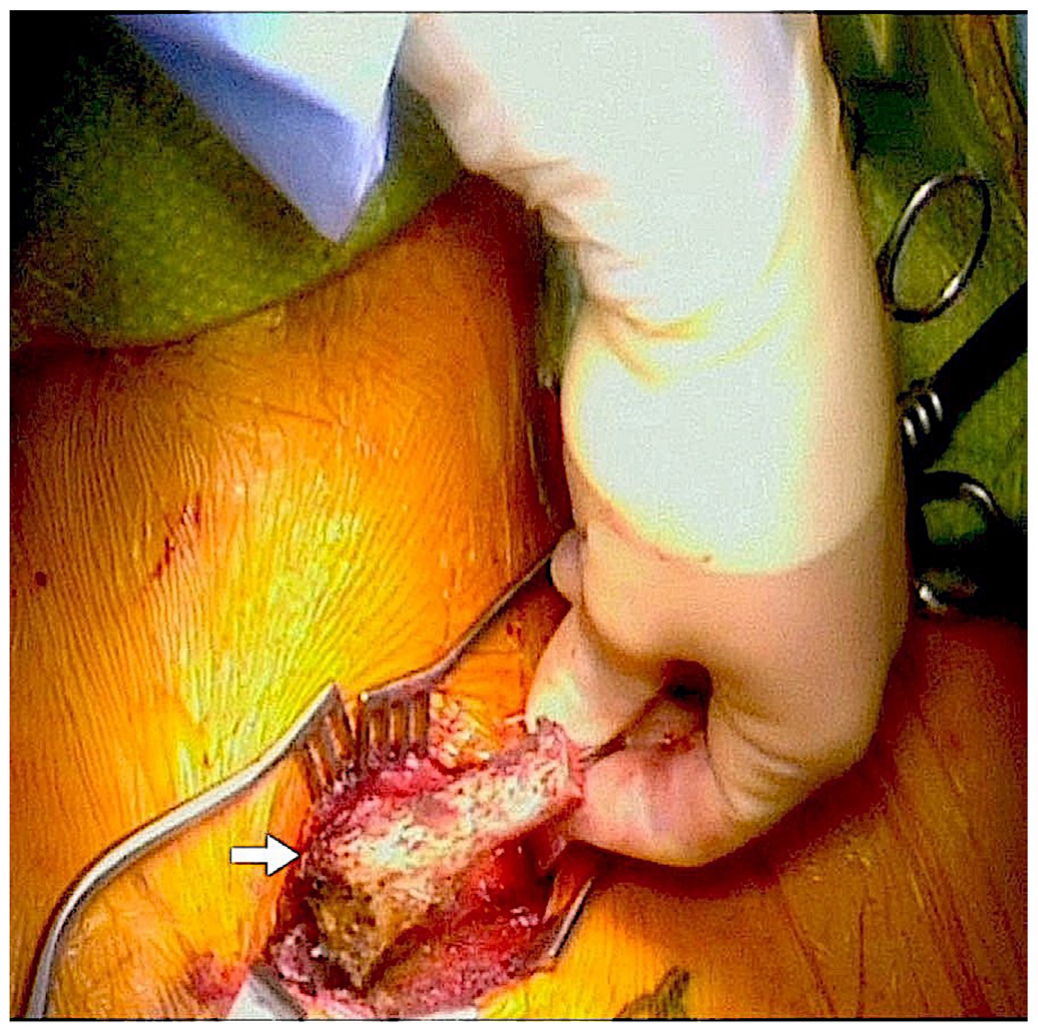
Intraoperative claviculectomy and removal of the medial two thirds of the clavicle (*arrow*).

**Fig 3:**
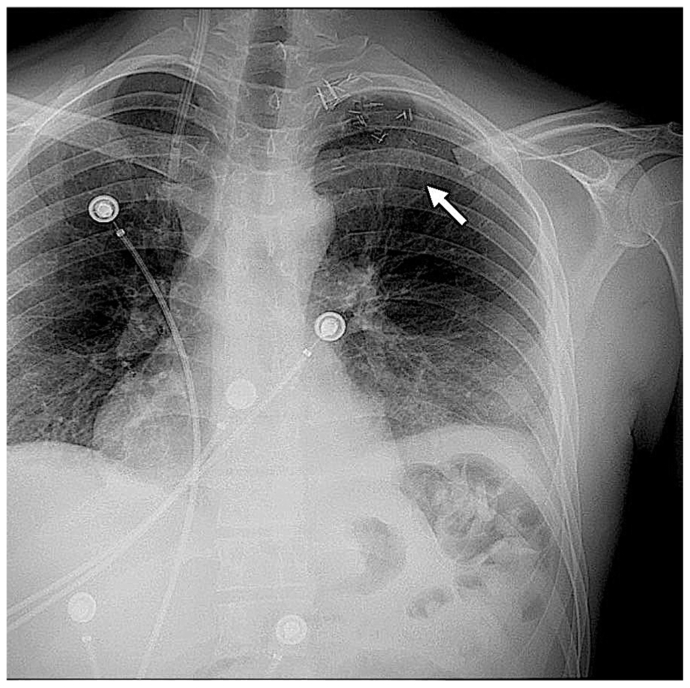
Postoperative day 3 chest radiograph demonstrating the resected clavicle and claviculectomy results (*arrow*).

Intraoperatively, an incision was made over the medial head of the clavicle and extending over approximately two thirds of the clavicle distally. The dissection was carried down, and the muscular attachments of the clavicle were dissected free cranially and caudally. The entire medial two thirds of the clavicle was dissected out circumferentially, the clavicle was transected using an oscillating saw, and the medial attachments were dissected off the sternum and removed (Fig 2). Next, major structures, such as the left brachiocephalic vein, left internal jugular vein, left subclavian vein, and thoracic duct, were identified. The anterior scalene muscle was divided while protecting the phrenic nerve to identify the subclavian artery at the thoracic outlet. The vagus nerve and carotid artery were also identified and mobilized appropriately. The subclavian artery, just proximal to the vertebral artery, left vertebral artery, and proximal axillary artery were identified and encircled with vessel loops. The left subclavian artery, left vertebral artery, and left axillary artery were all clamped. The mycotic aneurysm was resected, and the subclavian artery was excised up to its patent portion at the level of the axillary artery. The intraoperative cultures did not show evidence of bacterial infection, most likely secondary to long-term antibiotic use and/or biofilm (at our institution, sonication or other methods are not used to break down biofilm). The proximal anastomosis was performed using the great saphenous vein to the subclavian artery just distal to the origin of the vertebral artery, and the distal anastomosis was performed onto the axillary artery. The bypass was flushed appropriately. At this time, the patient had a palpable left radial artery and appropriate Doppler signals. The patient was extubated, transferred to the postanesthesia care unit, and discharged 3 days later in stable condition with antiplatelet and anticoagulation therapy and without any restricted shoulder range of motion (Fig 3). The patient was subsequently prescribed a postoperative antibiotic with the presumption of an infectious etiology, given the patient's history.

## Discussion

Subclavian artery aneurysms are rare, accounting for <0.15% of all aneurysms.^7^^,^^8^ Several etiologies contribute to the formation of subclavian artery aneurysms; however, they can be divided into two major categories: noninfectious and infectious (mycotic).^7^ Endocarditis, drug abuse, septicemia, poorly controlled soft tissue infection, and an immunocompromised state can lead to the formation of a mycotic aneurysm.^7^^,^^9^ Compared with noninfected aneurysms, mycotic aneurysms are extremely rare, can be due to direct invasion of the vessel walls from existing infections, and are, ultimately, associated with poor prognosis.^7^^,^^10^ As such, all SAMAs must be treated in a timely manner to prevent further complications such as hemorrhage, worsening septicemia, and arteriovenous fistula creation.^1^ Broad-spectrum intravenous antibiotics, followed by specific antibiotics based on cultures, should also be initiated and maintained for a minimum of 6 weeks, depending on the mycotic aneurysm characteristics and type of repair.^1^ To this day, open surgical treatment remains the gold standard as the most conventional repair modality for SAMAs.^1^^,^^7^ Because of the infectious nature of SAMAs, excision of all infected tissue is imperative in preventing reinfection and ensuring better postoperative outcomes for these patients.^3^^,^^11^ In contrast, for noninfectious subclavian artery aneurysms, endovascular, open, and hybrid approaches have had similar morbidity and mortality rates.^2^^,^^4^^,^^5^^,^^12^^,^^13^

Access options and the various methods of exposure for open repair of a SAMA depend greatly on the location of the aneurysm and the intra- or extrathoracic involvement.^2^^,^^4^^,^^5^ For proximal subclavian artery aneurysms, the method of exposure includes, but is not limited to, thoracotomy, sternotomy, and supraclavicular access. For the distal two thirds segment of the subclavian artery, supraclavicular exposure is often used.^2^ Nevertheless, claviculectomy is rarely used as the method of exposure for SAMAs. Despite the lack of use of claviculectomy in SAMA access, this is an effective treatment for diseases affecting the sternoclavicular joint.^4^^,^^14^ Patients with an excised medial clavicle maintain an unimpeded shoulder mobility without any significant deformities or cosmetic shortcomings.^4^^,^^14^^,^^15^ Compared with claviculectomy, which has no clear contraindications, other methods of exposure such as thoracotomy or sternotomy have an increased risk of postoperative pulmonary and esophagopleural complications, especially in already vulnerable patients.^2^ In the present case, the comorbidities associated with a high-risk patient directed the repair away from more invasive methods, such as thoracotomy or sternotomy, and toward the alternative method of claviculectomy to prevent intra- and postoperative complications. The mycotic etiology of the aneurysm further directed the repair. Endovascular methods were avoided due to the need to completely excise the infected aneurysm. Understanding the etiology and location of the aneurysm, paired with the comorbidities associated with the patient, resulted in our the successful decision to repair the SAMA using a median claviculectomy.

## Conclusions

SAMAs are rare and are often associated with endocarditis or intravenous drug use. SAMAs should be treated in a timely manner, and the size and location of the aneurysm should be considered to ensure proper surgical approaches to treatment. Claviculectomy can be effectively used to aid subclavian artery exposure by avoiding sternotomy or thoracotomy, depending on the location of the aneurysm and involvement from intra- or extrathoracic portions of the subclavian artery.

## Disclosures

None.
